# Calpain Inhibitor Calpeptin Alleviates Ischemia/Reperfusion-Induced Acute Kidney Injury *via* Suppressing AIM2 Inflammasome and Upregulating Klotho Protein

**DOI:** 10.3389/fmed.2022.811980

**Published:** 2022-01-28

**Authors:** Yong Wu, Huan Yang, Ming Cheng, Jialin Shi, Weichen Zhang, Shaojun Liu, Minmin Zhang

**Affiliations:** Department of Nephrology, Huashan Hospital and Nephrology Institute, Fudan University, Shanghai, China

**Keywords:** acute kidney injury, calpain, calpeptin, AIM2 inflammasome, Klotho

## Abstract

Renal ischemia/reperfusion injury is a major contributor of acute kidney injury (AKI), leading to renal cell necrosis, apoptosis, and inflammation. Calpains, a family of Ca^2+^-dependent cysteine proteases, play a pivotal role in the pathogenesis of renal diseases. Several studies have reported calpain inhibitors showing remarkable reno-protective effects against proteinuria and α-klotho deficiency-induced renal aging symptoms, particularly against glomerulus injury. However, little is known about the role of the calpain inhibitor calpeptin in acute kidney injury. The present study aims to investigate the potential mechanism of downregulation of Calpain 1 and 2 activity by calpeptin in the ischemia/reperfusion (IR)-induced AKI model. Firstly, we observed that the contents of Calpain 1 and 2 were significantly increased in the renal biopsy of clinical AKI patients, especially in the diseased tubules space. To investigate the impacts of calpain activity inhibition, we further pretreated with calpeptin in both the IR mouse model and in the HK-2 cells hypoxia model. We found that the calpain inhibitor calpeptin improved renal functional deterioration, attenuated pathological structure damage, and decreased tubular cell apoptosis in the IR injury-induced AKI mice model. Mechanistically, calpeptin significantly suppressed the AIM2 (absent in melanoma 2) and NLRP3 (NOD-like receptor protein 3) inflammasome signaling pathways and increased Klotho protein levels. Furthermore, immunofluorescence assays demonstrated that the application of calpeptin effectively inhibited Calpain 1 activation and gasdermin D (GSDMD) cleavage in the renal tubules of IR mice. Taken together, our both *in vivo* and *in vitro* experiments suggest that calpeptin conveyed reno-protection in AKI might be mediated by the inhibition of AIM2 inflammasome activation and upregulation of Klotho protein. As such, we provide new evidence that Calpain 1 and 2 activation may be closely associated with the pathogenesis of clinical AKI. The calpain-mediated AIM2 inflammasome signaling pathway and distinct interaction between calpain and Klotho may provide a potential novel preventative and therapeutic target for acute kidney injury.

## Introduction

Acute kidney injury (AKI), which is defined as the rapid decline of renal function decline over a short period of time, poses a significant threat to public health (1). Renal ischemia/reperfusion (IR) injury, a major cause of AKI, results in overwhelming inflammation and mediates an immune response (2, 3). Patients, especially those undergoing kidney, cardiac, or liver transplants, are particularly vulnerable to clinical AKI (4). In fact, IR-induced AKI results in high hospitalization rates, morbidity, and mortality, but there remains few effective strategies for prevention or treatment because of the incomplete knowledge of the mechanism (5).

The absent in melanoma 2 (AIM2) inflammasome plays an important role in inflammatory and immune responses to endogenous pathogens and cellular perturbations (6, 7). In principle, AIM2 recognizes double-stranded DNA (dsDNA) from necrotic cellular debris and subsequently activates Caspase 1 and recruits an apoptosis-associated speck-like protein containing a CARD (ASC) to complete the inflammasome assembly, eventually triggering the maturation and release of IL-1β and IL-18 and promoting gasdermin-D (GSDMD) cleavage which induces pyroptosis (8, 9). Several studies have found that AIM2 inflammasome is closely associated with renal diseases, including lupus nephritis (10, 11) and hepatitis B-associated glomerulonephritis (12). Similarly, the role of the AIM2 inflammasome in renal interstitial injury has been shown in the unilateral ureteral occlusion (UUO) mouse model. Komada and colleagues found that renal resident macrophages that take up dsDNA from necrotic cells activate the AIM2 inflammasome signaling pathway, ultimately exacerbating UUO-induced renal fibrosis and inflammation; in contrast, the knockout of the AIM2 gene effectively reduces these effects (13). To date, studies have focused on the role of AIM2 inflammasome in the pathogenesis of chronic kidney disease (CKD); however, the explicit mechanism of the AIM2 inflammasome in AKI remains unknown.

Calpains are a family of calcium-dependent cytosolic cysteine proteases which includes calpain 1 (μ-form) and calpain 2 (m-form) (14, 15). It has been reported that calpains are regulated by Ca^2+^ in the cytoplasm and are involved in the modulation of cellular biological behaviors, inflammasome activation, and cell apoptosis *via* binding to the corresponding receptor or substrate (16). Notably, Louise and colleagues demonstrated that transient receptor potential channel 6 (TRPC6) regulates cell motility and detachment of podocytes through its interaction with calpain 1 and 2 (17, 18). Interestingly, calpeptin is the specific calpain inhibitor involved in cell penetration, significantly decreasing podocyte injury and reducing proteinuria levels in the adriamycin nephropathy mouse model of focal segmental glomerulosclerosis (FSGS) (17). Consistent with this, Tian et al. also found that calpain inhibitor III decreased the degree of proteinuria, alleviated glomerulosclerosis, and even improved survival rates in the podocyte-specific Gak-knockout mice through the specific inhibition of calpain 1 and 2 activities (19). Furthermore, Nabeshima et.al. revealed that daily administration of the calpain 1 inhibitor BDA410 decreased α-klotho deficiency-induced aging syndrome and maintained mineral homeostasis in CKD (20). Indeed, the available evidence suggests that calpain inhibitors have remarkable anti-aging, anti-inflammatory, and nephroprotective effects, especially with respect to glomerulus injury. However, the specific impacts of the calpain inhibitor calpeptin in acute tubular injury remain poorly understood. The aim of this study was to determine the effects of calpeptin on ischemia/reperfusion-induced AKI and to investigate its possible mechanisms.

## Methods and Materials

### Human Renal Biopsy Study

This study was approved by the Ethics Committee on Human Research of Huashan Hospital, Fudan University and all procedures were performed in accordance with the World Medical Association Declaration of Helsinki. Signed informed consent documents were obtained from all subjects before their participation. The renal biopsy samples and clinical medical records of AKI patients pathologically verified as acute tubular injury (ATI) (*n* = 10) were retrospectively collected and analyzed from the renal pathology database of Huashan Hospital, Fudan University from January 2020 to June 2021 (Table 1). AKI diagnosis was in accordance with KDIGO definition (21), and the exclusion criteria were other immunological factors-induced clinical AKI including lupus nephritis, ANCA (Anti-neutrophil cytoplasmic antibodies)-associated vasculitis, anti-GBM (Glomerular basement membrane) nephritis, and crescentic glomerulonephritis. Normal kidney tissue near renal carcinomas was used as the normal control group (*n* = 6). All human kidney sections were stained with Calpain 1 and Calpain 2 by immunohistochemistry and then viewed under a Leica microscope.

**Table 1:** Clinical parameters of AKI patients and normal controls.

**AKI**	**Gender**	**Age (years)**	**SCr (μmol/L)**	**BUN (mmol/L)**	**eGFR (ml/min/1.73 m^**2**^)**	**Urine protein (g/ 24 h)**	**Pathological diagnosis**
1	Female	54	196	16.2	24.4	7.22	ATI; Podocytopathy
2	Male	17	226	15.7	35.0	21.24	MCD; ATI
3	Male	26	182	9.2	43.2	<0.07	AIN; ATI
4	Female	62	116	9.2	57.8	12.66	FSGS; ATI; DN
5	Male	38	148	8.4	51.0	>25	ATI; MCD
6	Male	29	737	13.4	7.8	0.27	ATI
7	Male	26	308	5.7	22.7	0.19	FSGS; ATI; DN
8	Male	56	190	16.7	33.9	9.28	FSGS; ATI
9	Male	48	161	20.5	41.8	8.69	FSGS; ATI
10	Female	69	523	38.9	7.5	0.08	FSGS; ATI
**Control**	**Gender**	**Age (years)**	**SCr (μmol/L)**	**BUN (mmol/L)**	**eGFR (ml/min/1.73m** ^**2**^ **)**		
1	Female	54	47	3.6	127.3		
2	Male	61	54	5	142.6		
3	Male	66	75	4.4	96.1		
4	Male	37	59	4.6	122.9		
5	Female	65	51	5.2	97.2		
6	Female	67	60	6.3	90.9		

*AKI, acute kidney disease; SCr, serum creatinine; BUN, blood urea nitrogen; Cys-C, cystatin C; eGFR, estimated glomerular filtration rate; ATI, acute tubular injury; MCD, minimal-change disease; AIN, acute interstitial nephritis; FSGS, focal segmental glomerulosclerosis; DN, diabetic nephropathy*.

### Renal Ischemia/Reperfusion Injury Model

All animal experiments were approved by the Ethics Committee for Experimental Research at Fudan University (No.2020 Huashan Hospital JS-507). Male C57/B6J mice (8–10 weeks old, 22–25 g) were provided by Shanghai Jihui Laboratory Animal Care Company. Briefly, mice for the renal IR injury mouse model were subjected to a right nephrectomy with anesthesia, and then the left renal pedicle was clamped for 30 min to initiate renal ischemia followed by reperfusion. The sham-operated group was exposed to both renal pedicles. The animal body temperature was maintained at 36–37°C during this surgery. Additionally, all mice were injected vehicle or calpeptin (CP, 40 ug/mouse/day, MedChemExpress) intraperitoneally for 5 days of preventative treatment prior to the operation (**Figure 2A**). These animals were randomly divided into the following groups: (1) sham; (2) CP; (3) IR; (4) CP+IR (*n* = 8 for each group). All mice were killed 24 h after reperfusion, and kidney and serum samples were obtained for further analyses.

### Renal Injury Detection

Serum creatinine (SCr) and blood urea nitrogen (BUN) levels were measured by an automatic chemistry analyzer (Roche c702, KingMed Diagnostics) to evaluate the changes of renal function. mRNA levels of the AKI biomarker NGAL were measured with RT-PCR and protein expression of LCN2 were measured with Western blots to assess the degree of mouse kidney damage and cell injury, respectively.

### Renal Pathological Staining

The 4% paraformaldehyde-fixed renal issue was embedded in paraffin and cut into 4-μm sections for H&E staining to identify any changes to renal pathology. The degree of kidney injury was estimated by allocating a tubular injury score: 0, no damage; 1, 0–25%; 2, 25–50%; 3, 50–75%; 4, >75%.

### Cell Culture and Treatment

Immortalized human proximal renal tubular epithelial cell line (HK-2) cells were kindly provided by Prof. Jing Chen (Department of Nephrology, Huashan Hospital and Nephrology Institute, Fudan University). The HK-2 cells were cultured in DMEM/F12 (1:1) medium (Gbicol) supplemented with 10% fetal bovine serum (Sigma-Aldrich) and 1% penicillin-streptomycin (Gbicol) at 37°C with 5% humidified carbon dioxide. HK-2 cells were incubated with 300 μM cobaltous chloride (CoCl_2_, 7646-79-9, Sigma-Aldrich) for 24 h to induce a hypoxia model. Half cells were pretreated with calpain inhibitor 20 μM calpeptin for 24 h before hypoxia intervention.

### Western Blot Analysis

Renal tissue and HK-2 cells total protein was extracted in RIPA lysis buffer (PC101, Epizyme) with 1% PMSF (ST506, Beyotime) and 1% protease inhibitor cocktail (HY-K0010, MedChemExpress). The concentration of each protein sample was measured with the enhanced BCA protein assay kit (Beyotime) according to the manufacturer's protocol. Each protein sample (40–50 ug) was run on an SDS-PAGE gel using a 10 or 12.5% PAGE gel fast preparation kit (Epizyme) and then transferred to a 0.22 μm PVDF membrane (Milipore). The membrane was blocked by protein free rapid blocking buffer (PS108P, Epizyme) for 20 min and then incubated overnight with primary antibodies against Calpain 1 (1:1000, #2556, Cell Signaling Technology), Calpain 2 (1:1000, # DF7807, Affinity), Klotho (1:500, # DF10309, Affinity), AIM2(1:500, # DF3514, Affinity), NLRP3 (1:500, # DF7438, Affinity), pro-Caspase 1 (1:500, #24232, Cell Signaling Technology), cleaved-Caspase 1 (1:500, #89332, Cell Signaling Technology), ASC (1:1000, sc-514414, Santa Cruz), IL-1β (1:500, #83186, Cell Signaling Technology), IL-18 (1:500, # DF6252, Affinity), LCN2 (1:500, #DF6816, Affinity), and GAPDH (1:10000, ab181602, Abcam). The membrane was then incubated with goat anti-rabbit or goat anti-mouse secondary antibody (1:15000, 926-68071, 926-6870, LI-COR) at room temperature away from light for 75 min. Bands were detected by the Odyssey Fc Imaging System (LI-COR Bioscience), and intensity values were analyzed with the Image J software.

### RNA Extraction and RT-PCR

Mouse kidney total RNA was isolated with Trizol reagent (Thermo scientific) and then reverse transcribed into cDNA according to the manufacturer's procedures (EnzyArtisan, Shanghai, China). Relative mRNA quantity was measured with the SYBR green mix kit (EnzyArtisan). Primer sequences of both target genes and house-keeping genes for RT-PCR are listed in the (Supplementary Table 1).

### Calpain and Cathepsin B Activity Detection

The calpain and cathepsin B activity of renal issues and HK-2 cells were respectively detected by using the calpain activity assay kit (ab65308, Abcam) and cathepsin B activity assay kit (ab65300, Abcam) according to the manufacturers' instructions.

### Immunohistochemistry and Immunofluorescence Staining

After deparaffinization and rehydration, renal slides were subjected to antigen retrieval with EDTA buffer through microwave heating. The slides were then blocked by 5% BSA for 1 h, and subsequently incubated overnight with a primary antibody against Calpain 1 (1:200, #DF6306, Affinity), Calpain 2 (1:200, # DF7807, Affinity), AIM2(1:200, # DF3514, Affinity), and GSDMD (1:200, sc-393581, Santa Cruz) at 4°C. After incubation with the secondary antibody, kidney sections were observed under a fluorescence microscope (Leica).

### TUNEL Assay

The kidney tissue sections were assessed to determine cell apotosis by TUNEL Apoptosis Assay Kit (Beyotime) following the manufacturer's protocols. The cell nucleus was stained with blue fluorescence with DAPI, and apoptotic cells were marked with red fluorescence. The number of TUNEL-positive cells were detected at least six random areas per slide with a fluorescence microscope (Leica).

### Statistical Analysis

All results were presented as mean ± SEM. All experimental data were analyzed with SPSS version 23.0 (IBM Corp.,) and GraphPad Prism version 8.0 (San Diego, CA). Multiple comparisons between the various groups were assessed using one-way analysis of variance (ANOVA) and Newman-Keuls *post-hoc* multiple range tests. Comparisons between two groups were assessed using Student *t*-test. A *p*-value of <0.05 was considered significant.

## Results

### The Contents of Calpain 1 and Calpain 2 Were Increased in the Renal Biopsy of AKI Patients

**Figure 1:**
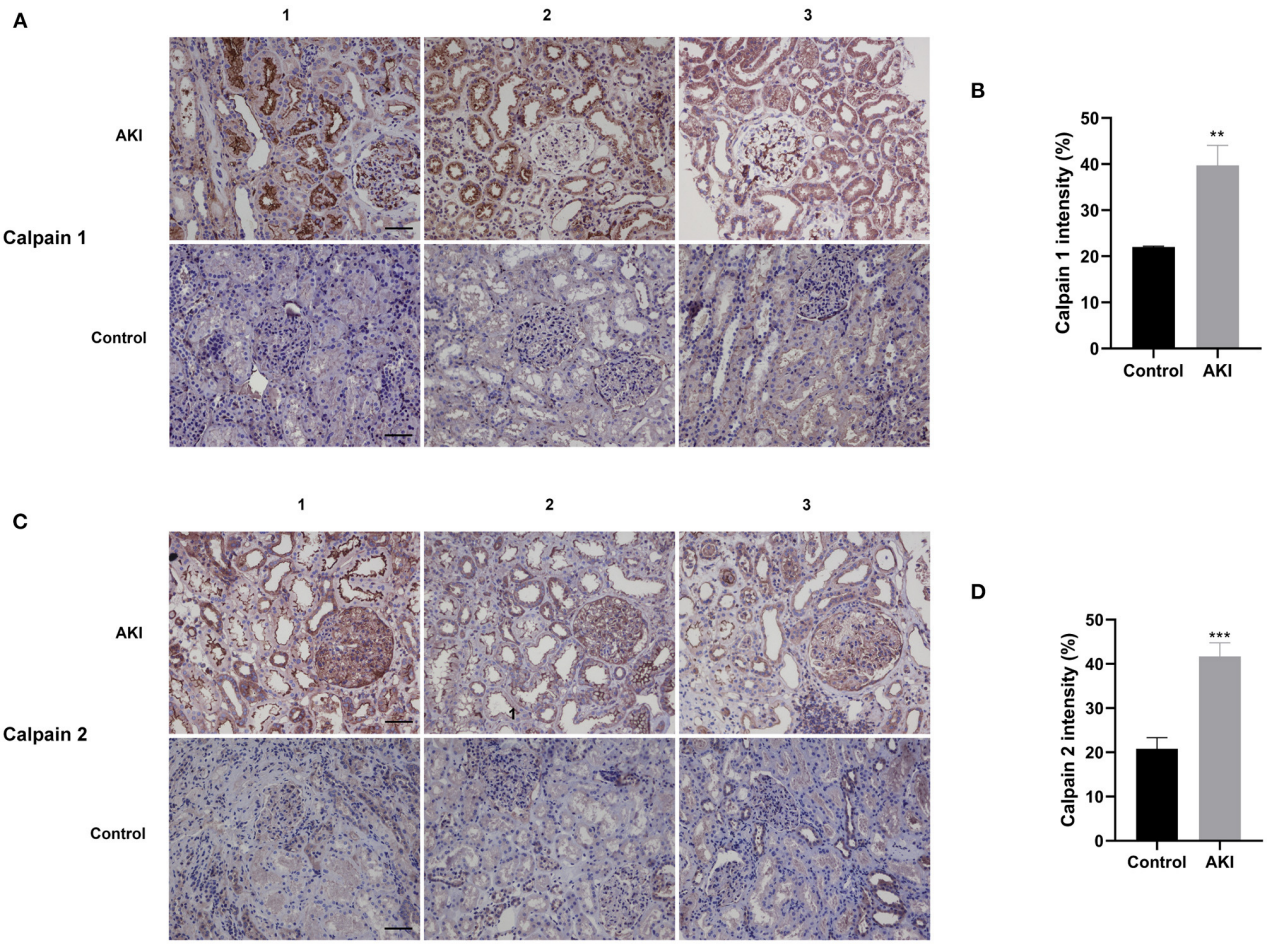
Calpain 1 and 2 are markedly increased in the renal biopsy of AKI patients. Representative immunohistochemical micrographs of Calpain 1 **(A)** and Calpain 2 **(C)** protein expression in the renal biopsy of both AKI patients (*n* = 10) and normal control group (*n* = 6). Quantitative analysis of Calpain 1 **(B)** and Calpain 2 **(D)** expression in the human renal specimens. ×200, bar = 100 μm. Data were presented as mean ± SEM (*n* = 3). **p < 0.01; ***p < 0.001 vs. normal control group. AKI, acute kidney injury.

To determine the distribution and expression of calpains in renal tissues, we collected and analyzed human renal biopsies by using immunohistochemical staining for Calpain 1 and Calpain 2 protein. Ten kidney biopsies with renal pathological diagnosis of acute tubular injury were enrolled in this study, while six normal renal specimens adjacent to renal carcinoma served as the normal control group. The clinical characteristics of AKI patients and normal controls were displayed in Table 1. The immunohistochemical staining result revealed that compared with normal control group, the contents of Calpain 1 (Figures 1A,B) and Calpain 2 (Figures 1C,D) protein were significantly increased among the kidney of AKI patients' group. Of note, Calpain 1 and Calpain 2 were predominantly distributed in the tubules space of the diseased kidneys. These findings suggest the novel clinical evidence that Calpain 1 and Calpain 2 induction may be closely linked to clinical AKI.

### Calpeptin Attenuates Renal Dysfunction and Renal Tubule Injury in IR Mouse Model

**Figure 2:**
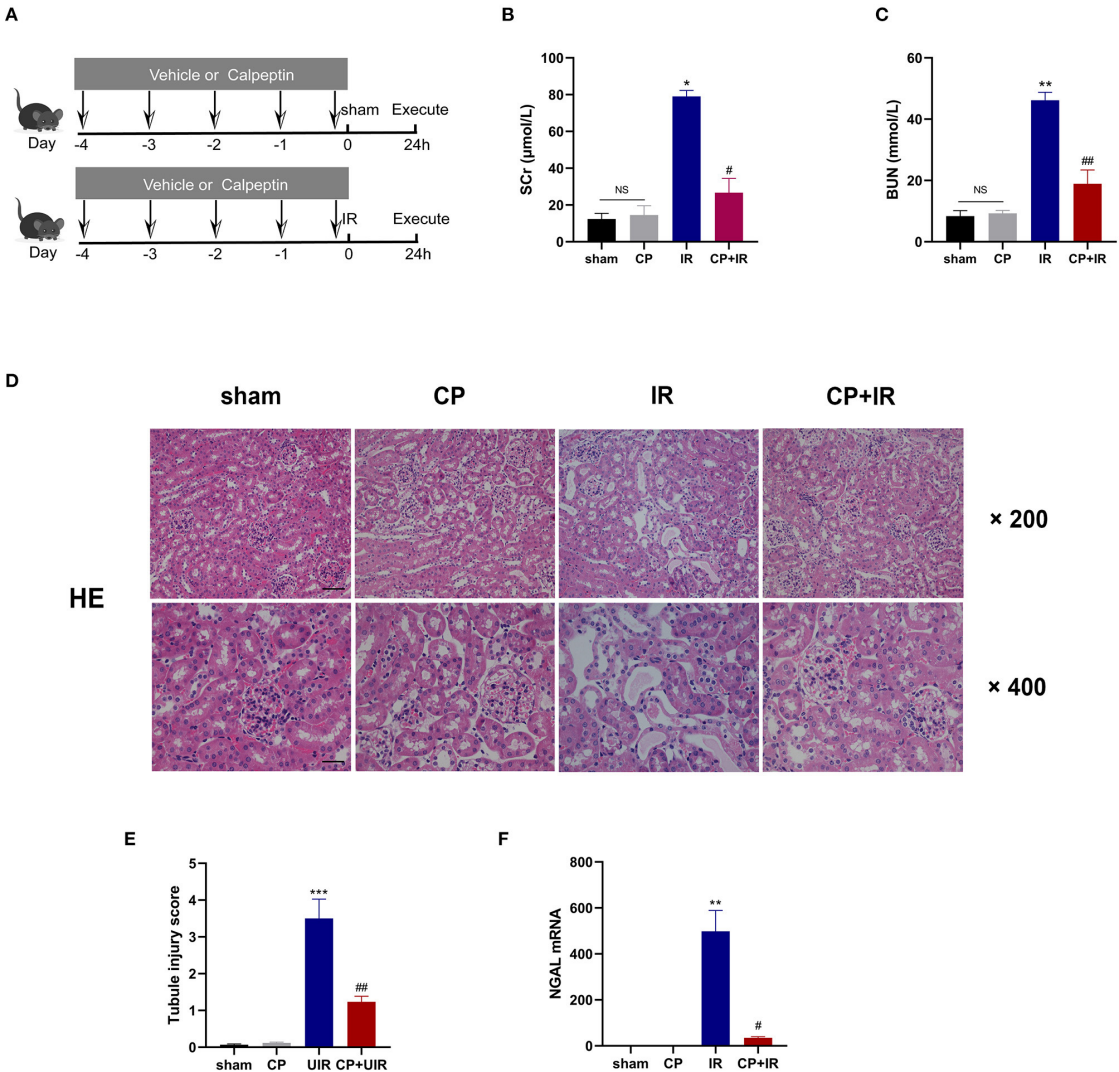
Calpeptin significantly ameliorates renal dysfunction and renal tubule injury in IR mouse model. **(A)** The schematic diagram of animal experiments was displayed and all mice were divided into 4 groups: (1) sham; (2) CP; (3) IR; (4) CP+IR. **(B,C)** The renal function was measured by serum creatine and blood urea nitrogen. **(D)** The renal pathological changes were assessed by H&E staining. ×200, bar = 100 μm; ×400, bar = 50 μm. **(E)** Quantitative analysis of tubule injury score. **(F)** The renal damages of all groups were evaluated by the NGAL mRNA levels. All values were presented as mean±SEM (*n* = 3). NS, no significance; *p < 0.05 vs. sham group; **p < 0.01 vs. sham group; ***p < 0.001 vs. sham group; #*p* < 0.05 vs. IR group; ##*p* < 0.01 vs. IR group; ###*p* < 0.001 vs. IR group. CP, calpeptin; IR, ischemia/reperfusion; SCr, serum creatine; BUN, blood urea nitrogen.

To investigate the role of Calpain in AKI, we established the IR mouse model and pretreated with the calpain inhibitor calpeptin before surgery. The detailed animal experimental procedures are shown in Figure 2A. Compared with sham group, serum creatinine and blood urea nitrogen levels were dramatically increased in the IR mice; however, the pretreatment with calpeptin significantly improved renal dysfunction (Figures 2B,C). As shown in the Figures 2D,E, pretreatment with calpeptin was found to reduce tubulointerstitial edema, lumen dilation, and brush-like margin abscission in the H&E staining. In lines with this, the AKI biomarker NGAL mRNA expression was significantly decreased in the CP+IR mice (Figure 2F). These results demonstrate that the inhibition of calpains with calpeptin attenuates renal dysfunction and tubular pathological damage in the IR-induced AKI mouse model.

### Calpeptin Inhibits AIM2 and NLRP3 Inflammasome Signaling Pathway, and Upregulates Klotho Protein Expression in IR Mouse Model

**Figure 3:**
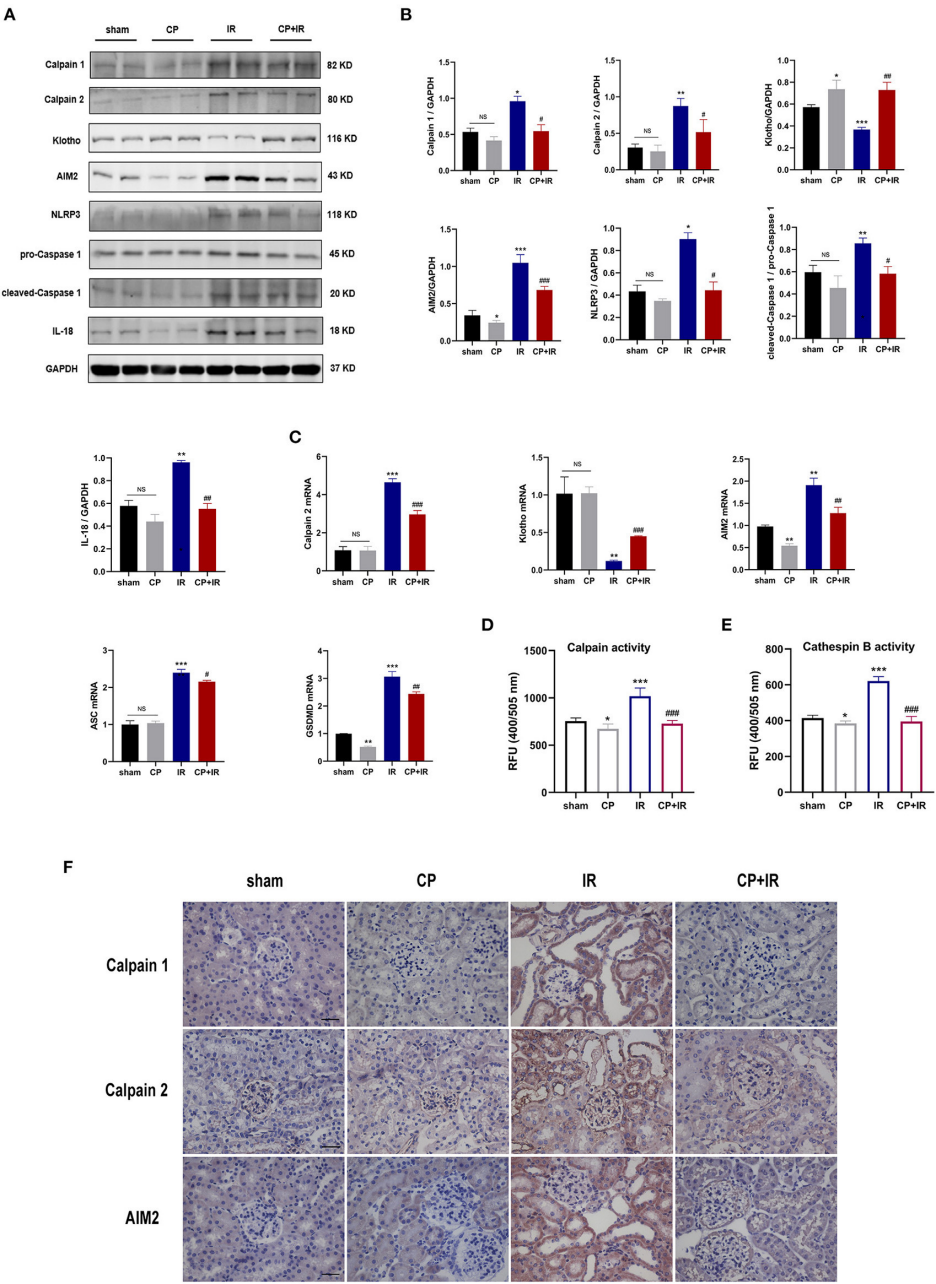
Calpeptin inhibits AIM2 and NLRP3 inflammasome-mediated inflammation, and upregulates Klotho protein expression in IR mouse model. **(A)** Western blotting of Calpain 1, Calpain 2, Klotho, AIM2, NLRP3, pro-Caspase 1, cleaved-Caspase 1, IL-1β and IL-18 in the kidney of all mice. **(B)** Quantitative determination of Calpain 1, Calpain 2, Klotho, AIM2, NLRP3, cleaved-Caspase 1 and IL-18. **(C)** The mRNA levels of Calpain 2, Klotho, AIM2, ASC and GSDMD in the kidney among different groups. **(D)** The calpain activity of renal issues was measured by the relative fluorescence units (400/505 nm). **(E)** The cathepsin B activity of kidney issues was tested by the relative fluorescence units (400/505 nm). **(F)** Representative immunohistochemical micrographs from the kidney issues of different groups stained with Calpain 1, Calpain 2 and AIM2. ×400, bar = 50 μm. All data were presented as mean ± SEM (*n* = 3). NS, no significance; *p < 0.05 vs. sham group; **p < 0.01 vs. sham group; ***p < 0.001 vs. sham group; #*p* < 0.05 vs. IR group; ##*p* < 0.01 vs. IR group; ###*p* < 0.001 vs. IR group. CP, calpeptin; IR, ischemia/reperfusion; RFU, relative fluorescence units.

**Figure 4:**
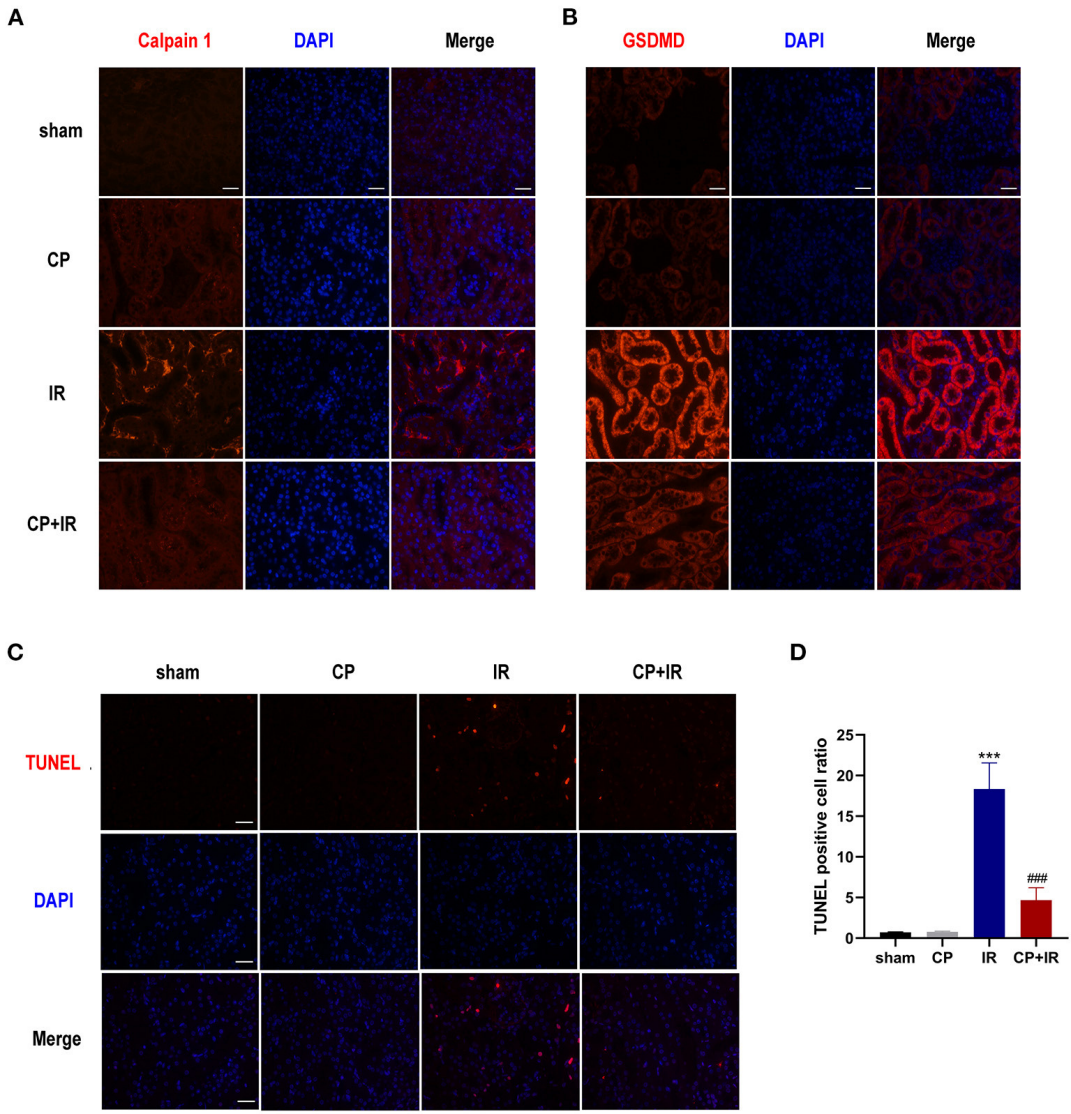
Calpeptin inhibits Calpain 1 activation and GSDMD cleavage in the tubules of IR mice, reduces cells apoptosis as well. **(A,B)** Representative immunofluorescence micrographs from mice kidneys of different groups stained Calpain 1 and GSDMD. ×400, bar = 50 μm. **(C)** Representative TUNEL staining micrographs in the mice kidneys of different groups. ×400, bar = 50 μm. **(D)** Quantitative determination of apoptosis cells amount. All Values were presented as mean ± SEM (*n* = 3). ***p < 0.001 vs. sham group; ###*p* < 0.001 vs. IR group. CP, calpeptin; IR, ischemia/reperfusion.

To further explore the possible mechanism of calpain inhibitor calpeptin, RT-PCR and western blotting were used to detect the effects on Calpain and Klotho expression, and on the AIM2 and NLRP3 inflammasome signaling pathways. As shown in the Figures 3A,B,D,E, there were marked increases in both Calpain 1 and Calpain 2 protein and the total calpain and cathepsin B activity among the kidney issues of IR mice. Additionally, the immunohistochemistry staining assay also confirmed that there was markedly induced expression of Calpain 1, Calpain 2 and AIM2 protein in the diseased tubules among the IR mice (Figure 3F). However, these inducible effects were significantly blocked by calpeptin (Figures 3A,B,D,E). Although the Klotho protein levels and mRNA levels showed no significant difference between CP group and sham group, there was a significant increase in Klotho mRNA and protein levels in the CP+IR mice compared with the IR mice (Figures 3A–C). We found that the inflammatory mediators in the kidney, AIM2, NLRP3, cleaved-Caspase 1, and IL-18, all showed significantly increased protein levels in the IR group compared with the sham group, although these changes were eliminated when pretreatment of calpeptin was administered (Figures 3A,B). The mRNA changes of AIM2, ASC, GSDMD and Calpain 2 were consistent with the protein levels (Figure 3C). Also, immunofluorescence assay showed that calpeptin significantly weakened the immunofluorescence intensity of Calpain 1 and GSDMD in the tubules compared with IR mice (Figures 4A,B). These findings suggest that calpeptin mitigates IR-induced renal damage by inhibiting AIM2 and NLRP3 inflammasome-mediated inflammation and upregulating Klotho protein.

### Calpeptin Reduces the Apoptosis of Renal Tubular Epithelial Cells in IR Mouse Model

Apoptosis is one of the major contributors to IR-induced AKI (21). We conducted TUNEL staining to determine the impacts of calpeptin on IR-induced cell apoptosis. More TUNEL-positive cells were observed in the IR mice kidneys than in the sham group; however, there was no significant difference between the sham group and the CP group. Importantly, fewer TUNEL-positive cells were viewed in the CP + IR mice kidneys than in the IR group (Figures 4C,D). These results suggest that calpeptin attenuates the amount of tubular epithelial cell apoptosis in the IR mouse model.

### Calpeptin Alleviates CoCl_2_-Induced HK-2 Cells Hypoxia Injury Through Blocking AIM2 and NLRP3 Inflammasome Activation and Upregulating Klotho Protein

**Figure 5:**
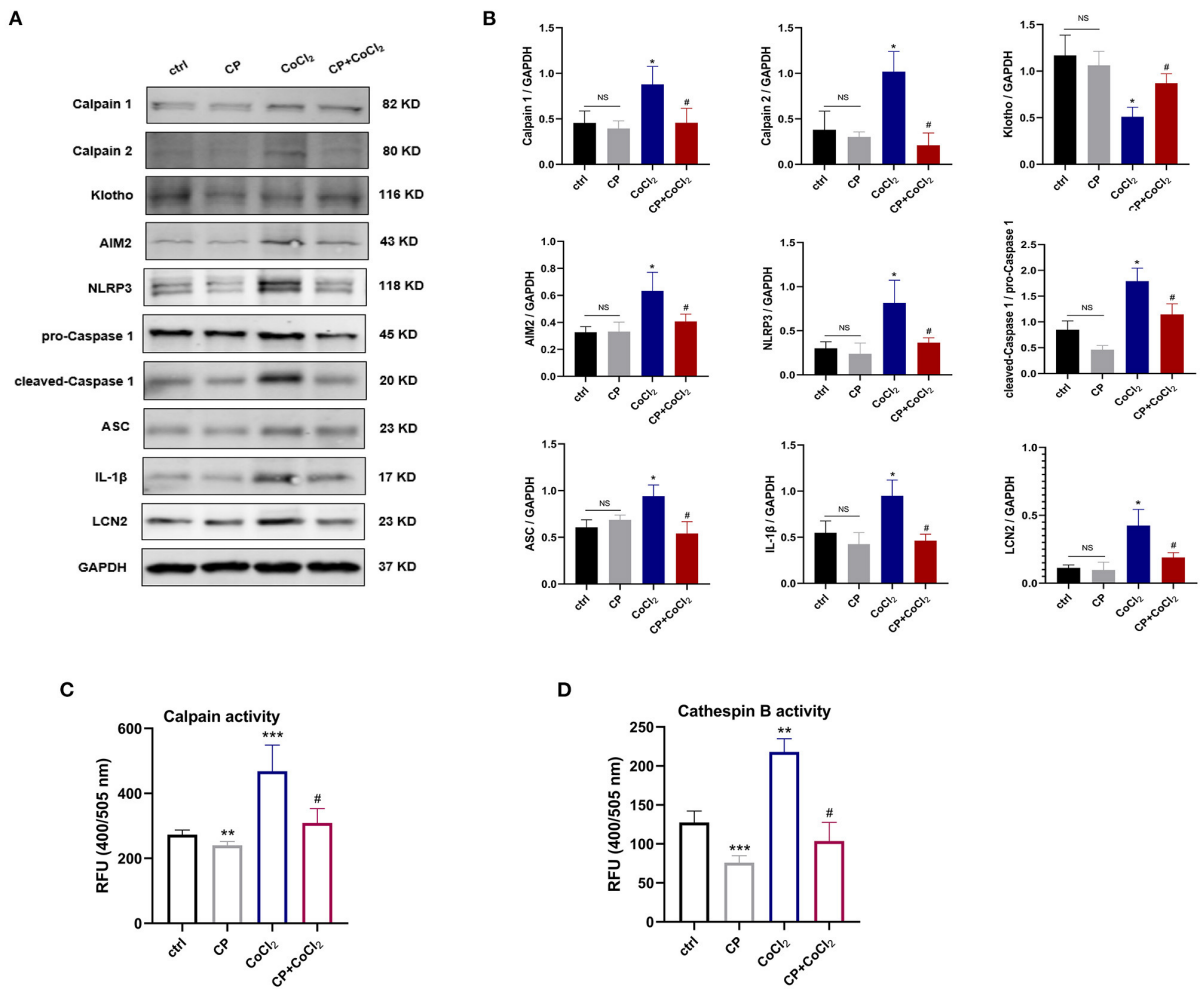
Calpeptin suppresses AIM2 and NLRP3 inflammasome signaling pathway, and increases Klotho protein expression in CoCl_2_-induced HK-2 cells hypoxia model. **(A)** Western blotting of Calpain 1, Calpain 2, Klotho, AIM2, NLRP3, pro-Caspase 1, cleaved-Caspase 1, ASC, IL-1β and LCN2 in the HK-2 cells. **(B)** Quantitative determination of Calpain 1, Calpain 2, Klotho, AIM2, NLRP3, cleaved-Caspase 1, IL-1β and LCN2. **(C)** The calpain activity of HK-2 cells was assessed by the relative fluorescence units (400/505 nm). **(D)** The cathepsin B activity of HK-2 cells was analyzed by the relative fluorescence units (400/505 nm). All data were presented as mean ± SEM (*n* = 3). NS, no significance; *p < 0.05 vs. control group; **p < 0.01 vs. control group; ***p < 0.001 vs. control group; #*p* < 0.05 vs. CoCl_2_ group; ##*p* < 0.01 vs. CoCl_2_ group; ###*p* < 0.001 vs. CoCl_2_ group. CP, calpeptin; RFU, relative fluorescence units.

To examine the role of calpeptin in the *in vitro* experiments we coincubated HK-2 cells with 300 μM CoCl_2_ to induce the cell hypoxia model of AKI. As shown in the Figures 5A–D, the calpain and cathepsin B activity and the protein levels of Calpain 1, Calpain 2, AIM2, NLRP3, cleaved-Caspase 1, ASC, IL-1β, and LCN2 were significantly increased in the CoCl_2_ group compared with the control group. When pretreated with calpeptin, these effects were significantly reduced. The Klotho protein expression changes between groups were similar to those *in vivo* experiments (Figures 5A,B). These findings suggest that calpeptin alleviates CoCl_2_-induced HK-2 cell hypoxia injury through the inhibition of the AIM2 and NLRP3 inflammasome signaling pathways and upregulation of Klotho protein.

## Discussion

Calpains are intracellular Ca^2+^-dependent cysteine proteases which are involved in renal injury diseases (22). Calpain 1 and Calpain 2 activities are closely linked to both renal inflammatory response and aging, and suppression of Calpain 1 and 2 has been shown to relieve glomerulosclerosis and proteinuria (19). However, the function of Calpain 1 and 2 activity in acute tubule injury remains unclear. In this research, we established an ischemia/reperfusion injury-induced AKI model and investigated the potential role of Calpain 1 and 2.

We first compared the distribution and expression of Calpain 1 and 2 proteins in the renal biopsies of clinical AKI patients and a normal control group to better clarify the association between calpains and AKI. We observed that Calpain 1 and 2 proteins were predominantly upregulated in the diseased tubules among AKI patients, suggesting that overactivation of Calpain 1 and 2 occurred as part of AKI. Given this finding, we then down regulated the activities of Calpain 1 and 2 through the application of the calpain inhibitor calpeptin to determine its possible mechanisms during both *in vivo* and *in vitro* experiments. Notably, we found that pretreatment with calpeptin effectively attenuated renal dysfunction and histopathological lesions, and decreased renal tubular cell apoptosis in IR mice. As such, the application of calpeptin may provide an effective medical strategy to treat AKI through down regulation of calpain activity.

It has been reported that the cell-penetrative calpain inhibitor calpeptin not only suppresses calpain activity, but also inhibits cathepsin, papain and activates rho-kinase (23–27). To our knowledge, Tang et al. has demonstrated that as the important lysosomal protease, cathepsin B was markedly activated in the mouse renal ischemia/reperfusion injury, while hydroxychloroquine administration could decrease the activity of cathepsin B, and attenuate renal damage and dysfunction (28). Similarly, we also observed that cathepsin B activity was significantly upregulated in both CoCl_2_-simulated HK-cells and IR mice kidney issues. However, the pretreatment of calpeptin effectively blocked this induction, which suggested that the downregulation of cathepsin B activity by drugs application may be an indispensable therapeutic target for the kidney ischemia/reperfusion injury. Thus, we cannot rule out the other possible effects of calpeptin in the present study, and further studies are still needed to investigate other potential protease signaling pathway mediated by calpeptin in the renal ischemia/reperfusion injury. In fact, Calpain 1 and 2 protein exist in both cytosol and mitochondria (29). Numerous studies have demonstrated that calpains in myocardial mitochondria were over activated in the course of myocardial ischemia/reperfusion injury, resulting in mitochondrial function impairment and energy metabolism disorder, even exacerbating myocardial infarction (30). Importantly, calpeptin administration also reduced apoptosis of myocardial cells, decreased infarct size and improved myocardial dysfunction (31). Moreover, a recent research has found that selective inhibition of calpain in myocardial mitochondria effectively inhibited mitochondrial ROS induction and reduced hypoxia/reoxygenation-induced rat myoblast cell death (32). These findings suggest that mitochondrial calpains activation plays a pivotal role in the ischemia/reperfusion injury of the heart. However, to date, the role of mitochondrial calpains in the renal ischemia/reperfusion injury was poorly elucidated. Hence, further studies are required to focus on the potential mechanism of mitochondrial calpains in ischemia/reperfusion-induced acute kidney injury.

Calpain activation also plays a critical role in inflammasome activation which mediates the maturation and secretion of a series of pro-inflammatory cytokines. Välimäki et al. found that ATP stimulation-induced NLRP3 inflammasome signaling pathway activation depended on calpain activity in human macrophages, and that calpain activity was also required for the activation of unconventional vesicle-mediated protein release (33). These findings show that calpains serve as an upstream activator of NLRP3 inflammasomes in human macrophages with ATP exposure. Similarly, we found that the administration of calpeptin effectively inhibited the AIM2 and NLRP3 inflammasome-mediated inflammatory response and reduced the release of IL-18 and IL-1β, suggesting that calpain may be an upstream regulator of AIM2 inflammasome during the course of acute kidney injury. AIM2 is an important regulator of innate immunity; it detects dsDNA in necrotic cell debris which activates cleaved-Caspase 1 (p20) and recruits ASC speck, eventually reaching the inflammasome assembly and mediating a series of pro-inflammatory cytokines to mature and release (34). However, the role of AIM2 inflammasome activation in acute kidney injury remains unclear. Kidney ischemia/reperfusion injury is accompanied by significant tubular cell necrosis and apoptosis. As such, the dsDNA released by necrotic tubules may be the crucial initial promotor of AIM2 inflammasome activation. Additionally, it has been reported that AIM2 inflammasome also affects the infiltration of macrophages in the kidney, aggravating the progress of lupus nephritis (10) and UUO-induced renal fibrosis (13). These results suggest that the AIM2 inflammasome signaling pathway may exert a critical effect on renal immune and inflammatory diseases. Further studies need to better understand the detailed mechanism between Calpain activity and the AIM2 inflammasome signaling pathway in IR-induced acute tubule injury. A recent study showed that during the process of inflammasome activation, GSDMD cleavage led to perforation of the plasma membrane and Ca^2+^ influx which also promoted calpain-dependent maturation (35). Additionally, our findings found that calpeptin significantly suppressed the levels of GSDMD cleavage in the renal tubules of IR mice, suggesting an important mechanistic link between calpain and GSDMD cleavage. Moreover, due to the GSDMD cleavage modulated by other Caspase family members, including Caspase 3, 8, and 11 (36, 37), we cannot rule out the possibility that calpeptin has a direct or indirect inhibitory effect on these other Caspase proteins. In fact, Caspase 1 is a substrate of calpain, and calpain activity also affects Caspase 1 activation. Zhang et al. has found that activated calpain promoted a pool of Caspase 1 cleavage and release to modulate NLRP3 inflammasome signaling pathway (38). By contrast, calpain silencing would attenuate mice myocardial ischemia/reperfusion injury *via* down regulating the NLRP3/Caspase 1 axis (39). Similarly, we observed that inhibition of Calpain 1 and 2 activity with calpeptin pretreatment significantly inhibited the AIM2/Caspase 1 axis *in vivo* and *in vitro* experiments. As the pivotal member of inflammasome assembly, Caspase 1 activation is also closely regulated by calpain, and it determines the activation status of downstream inflammasome signaling pathways. Overall, these findings suggest that targeting the blockage of calpain may be a preventative and therapeutic measure when addressing inflammatory responses in the process of acute kidney injury.

Klotho is an anti-aging and anti-fibrosis protein that plays a crucial role in the physiology and pathophysiology of renal diseases (40). It has previously been reported that Klotho confers reno-protection against AKI in proximal tubular cells through an antioxidative effect (41). However, Klotho deficiency contributes to the degradation of cytoskeletal elements, which causes calpastatin, an endogenous inhibitor of calpain, to decrease, and leads to Calpain 1 over activation (42). Furthermore, Camilli et al. observed that Klotho reduced melanoma cell invasive potential by suppressing calpain expression and filamin cleavage (43). Interestingly, the calpain 1 inhibitor BD-410 significantly upregulates Klotho protein which ameliorates aging-induced syndromes and remedies calcium and phosphorus metabolism disorders (20). Likewise, we found that pretreatment with calpeptin in both IR mice and HK-2 cells treated with hypoxia also increased Klotho expression, suggesting that it can attenuate IR-induced AKI through the activation of the Klotho/Calpain 1 signaling pathway. IR injury-induced renal dysfunction was accompanied by an increase in the fractional excretion of Ca^2+^ and by an impairment in the balance of the fibroblast growth factor 23-klotho-vitamin D axis through down regulation of Klotho expression (44, 45). In fact, the activation of Calpain depends on the level of Ca^2+^. These results suggest that there is a distinctive interaction between both Klotho and Calpain 1 which may mediate renal inflammatory injury. Latanoprost, an ocular hypotensive drug, exerts a neuroprotective effect through Klotho promotion-mediated inhibition of Calpain activation, further supporting the remarkable interplay between Klotho and Calpain (46). Although calpeptin and Latanoprost act on different tissue and organ targets, both agents share similar pharmacological effects in response to kidney or nerve injury. Together, these findings show that the Klotho/Calpain 1 signaling pathway plays an indispensable role in acute renal injury. Additional studies are needed to investigate the detailed crosstalk between Klotho and Calpain 1 in other renal disease models.

In conclusion, this study shows that the inhibition of Calpain 1 and 2 activity by calpeptin plays a nephroprotective role in ischemia/reperfusion-induced acute kidney injury by suppressing AIM2 and NLRP3 inflammasome activation and by upregulating Klotho protein.

## Data Availability Statement

The original contributions presented in the study are included in the article/Supplementary Material, further inquiries can be directed to the corresponding author/s.

## Ethics Statement

The studies involving human participants were reviewed and approved by the Ethics Committee on Human Research of Huashan Hospital, Fudan University. The patients/participants provided their written informed consent to participate in this study. The animal study was reviewed and approved by the Ethics Committee on Human Research of Huashan Hospital, Fudan University. Written informed consent was obtained from the individual(s) for the publication of any potentially identifiable images or data included in this article.

## Author Contributions

YW, HY, and MC conducted the experiments. WZ and JS offered support about statistical analysis and experimental technology. SL and MC collected and analyzed clinical data of AKI patients. YW analyzed the data and wrote the manuscript. YW and MZ designed this research. MZ revised the manuscript critically for significant intellectual content. All authors contributed to the article and approved the submitted version.

## Funding

This work was financially supported by the National Natural Science Foundation of China (No. 81870501) and Shanghai Science and Technology Commission Fund (No. 19411967800).

## Conflict of Interest

The authors declare that the research was conducted in the absence of any commercial or financial relationships that could be construed as a potential conflict of interest.

## Publisher's Note

All claims expressed in this article are solely those of the authors and do not necessarily represent those of their affiliated organizations, or those of the publisher, the editors and the reviewers. Any product that may be evaluated in this article, or claim that may be made by its manufacturer, is not guaranteed or endorsed by the publisher.
