# Supplementary material for: Calpain Inhibitor Calpeptin Alleviates Ischemia/Reperfusion-Induced Acute Kidney Injury via Suppressing AIM2 Inflammasome and Upregulating Klotho Protein
Source: Front Med (Lausanne). 2022 Jan 28;9:811980. doi: 10.3389/fmed.2022.811980 (PMC8831790; doi:10.3389/fmed.2022.811980)

Supplementary material

Table S1. Mouse primers sequences of both target genes and house-keeping genes.

Gene	Forward sequence	Reverse sequence
**Calpain 2**	5’-TACCTTCCTGGTGGGTCTCATC-3’	5’-TTTGCCGAGGTGGATGTTGGTC-3’
**Klotho**	5’-AAGAACAACCTCTCGTCTCTTCTG-3’	5’-ATTGGCGGAACTTCATGTTAGG-3’
**AIM2**	5’-AGGCTGCTACAGAAGTCTGTCC-3’	5’-TCAGCACCGTGACAACAAGTGG-3’
**ASC**	5’-AGACCACCAGCCAAGACAAG-3’	5’-CTCCAGGTCCATCACCAAGT-3’
**GSDMD**	5’-GCGCTTTGTTCCATCGGAAAG-3’	5’-CCATTTCCAAGCTCTCCAGTTCTG-3’
**GAPDH**	5’-CATCACTGCCACCCAGAAGACTG-3’	5’-ATGCCAGTGAGCTTCCCGTTCAG-3’

Figure S1. Representative original images of Western blotting in **Figure 3A**.

Western blotting of Calpain 1, Calpain 2, Klotho, AIM2, NLRP3, pro-Caspase 1, cleaved-Caspase 1 and IL-18 in the kidney of all mice.

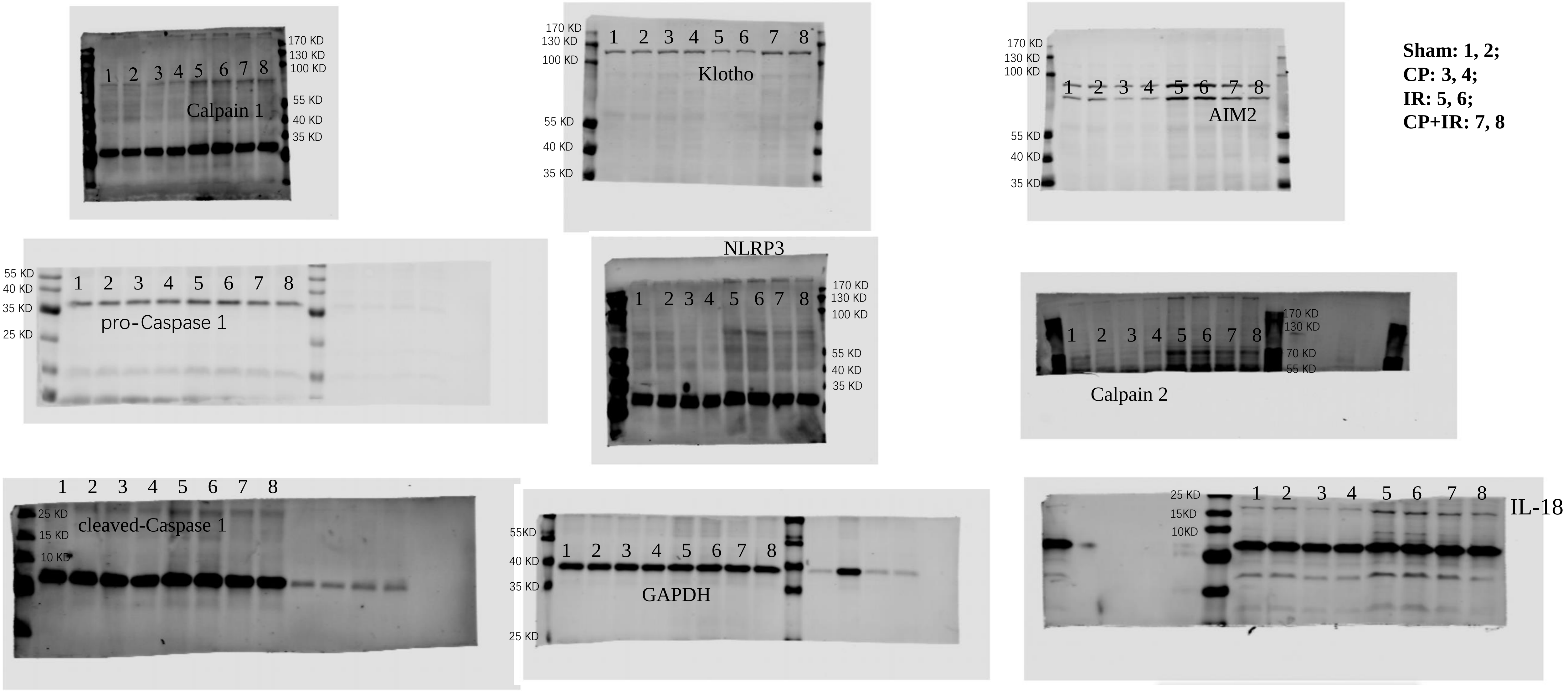

Sham: 1, 2;
CP: 3, 4;
IR: 5, 6;
CP+IR: 7, 8

Figure S2. Representative original images of Western blotting in **Figure 5A**.

Western blotting of Calpain 1, Calpain 2, Klotho, AIM2, NLRP3, pro-Caspase 1, cleaved-Caspase 1, ASC, IL-1β and LCN2 in the HK-2 cells.

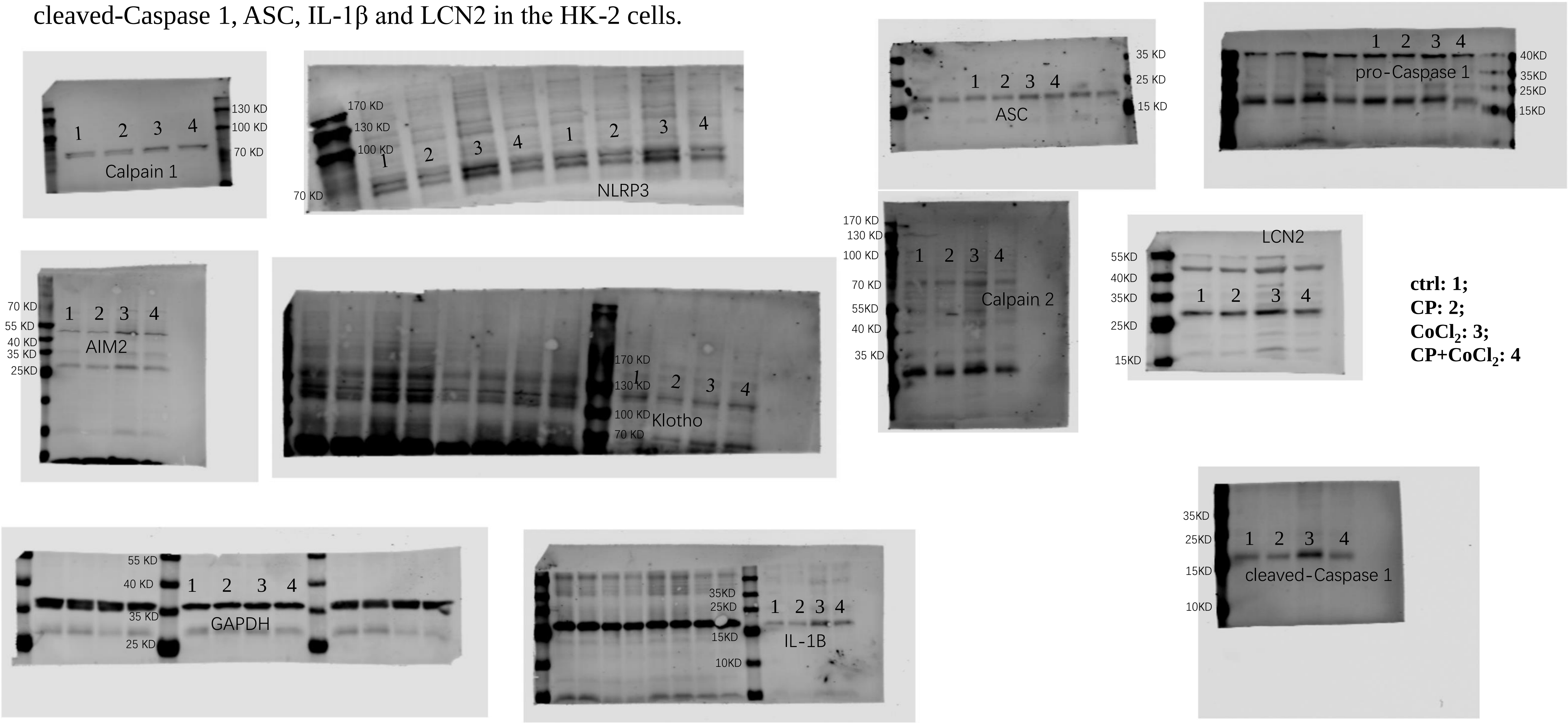

Supplement: Supplementary file 1 [file Data_Sheet_1.PDF]
